# The Role of Nrf2 in the Antioxidant Cellular Response to Medical Ozone Exposure

**DOI:** 10.3390/ijms20164009

**Published:** 2019-08-17

**Authors:** Mirco Galiè, Viviana Covi, Gabriele Tabaracci, Manuela Malatesta

**Affiliations:** 1 Department of Neurosciences, Biomedicine and Movement Sciences, University of Verona, Strada Le Grazie 8, I-37134 Verona, Italy; 2 San Rocco Clinic, Via Monsignor G. V. Moreni 95, I-25018 Montichiari (BS), Italy

**Keywords:** ozone therapy, oxidative stress, proteostasis, mitochondria, inflammation, adipose biology, cancer

## Abstract

Ozone (O_3_) is a natural, highly unstable atmospheric gas that rapidly decomposes to oxygen. Although not being a radical molecule, O_3_ is a very strong oxidant and therefore it is potentially toxic for living organisms. However, scientific evidence proved that the effects of O_3_ exposure are dose-dependent: high dosages stimulate severe oxidative stress resulting in inflammatory response and tissue injury, whereas low O_3_ concentrations induce a moderate oxidative eustress activating antioxidant pathways. These properties make O_3_ a powerful medical tool, which can be used as either a disinfectant or an adjuvant agent in the therapy of numerous diseases. In this paper, the cellular mechanisms involved in the antioxidant response to O_3_ exposure will be reviewed with special reference to the activation of nuclear factor erythroid 2-related factor 2 (Nrf2) and its role in the efficacy of ozone therapy.

## 1. Ozone in the Biomedical Field

Ozone (O_3_) is a natural gas forming from dioxygen (O_2_) by the action of ultraviolet light and electrical discharges in the atmosphere; however, O_3_ is highly instable, rapidly breaking down to its diatomic allotrope. For this reason, it occurs in very low amounts in the atmosphere compared to O_2_. O_3_ is known as a strong oxidant being at the same time a dangerous respiratory hazard and pollutant contributing to several diseases (recent review in [1,2,3,4,5]); it is also a powerful oxidizing agent with manifold industrial applications (e.g., as disinfectant, deodorizer, cleaning and bleaching agent) [6,7,8,9] and consumer implementations (e.g., as food additive, or air and water purifier) [10,11,12,13]. 

During the last decades, O_3_ has increasingly been applied as O_2_-O_3_ mixtures also in complementary/adjuvant medicine. Actually, the medical use of O_3_ dates back to the 18th century [14], when the Dutch physicist Martin van Marum discovered that an electric spark through the air gave rise to a gas with a typical odour and strong oxidizing properties. However, the link between dioxygen chemical modification and formation of the oxidant gas was only understood in 1840 by the German chemist Christian Friedrich Schönbein who called this gas “ozone” (from the Greek word ὄζειν, “to smell”) and revealed its capability to interact with organic compounds. Subsequently, various generators of O_3_-O_2_ mixtures were built, and the first studies on the biological effects of O_3_ were performed. Consequently, in the 19th century, O_3_ started to be used for therapeutic purposes, especially in treatment of allergy, and even Nikola Tesla patented an O_3_ generating system for medical use. During the last century, O_3_ was administered by different ways (gaseous O_3_ inhalation, injection or bags; auto-hemotherapy; ozonated water or oil) to treat a number of diseases (e.g., anaemia, diabetes, fever, gangrene, syphilis, tetanus, tuberculosis). Starting from late 1970s, O_2_-O_3_ therapy had a great development all over the world, thanks to the refinement of administration techniques and protocols, and to the collaborative work of some European medical societies. This contributed significantly to the set-up of regulatory requirements for O_3_ application, including the production of medical O_3_, the use of appropriate disposable materials, and the definition of concentration, dosages, and treatment frequency in relation to the disease [15]. In addition, based on clinical practice and according to the principle of “hormesis” (i.e., “the beneficial effect of a low level exposure to an agent that is harmful at high levels”) [16], a progressive lowering of O_3_ dosage has been adopted, relaying its beneficial effects to the cascade of metabolic events triggered by the initial, mild oxidative stress [17].

In parallel to clinical advancement, an increasing number of scientific reports have been published on the biomedical effects of O_3_ as adjuvant/alternative therapeutic approach for e.g., pain management [18,19,20,21,22], gastrointestinal diseases [23,24,25,26,27], lung diseases [28,29,30,31], diabetes [32,33,34,35,36,37], ischemia [38,39,40,41,42,43], cancer [44,45,46], infective diseases [47,48,49,50], dentistry [51,52,53,54,55,56].

However, despite the wide scientific demonstration of the multiple therapeutic applications of O_2_-O_3_ treatment, the biological mechanism(s) responsible for the positive effects of O_3_ administration have been only partially elucidated.

## 2. Ozone-Induced Activation of the Nuclear Factor Erythroid 2-Related Factor 2 (Nrf2)

Exposure to toxic levels of atmospheric O_3_ induces injury and inflammation through activation of the redox sensitive nuclear factor kappa-light-chain-enhancer of activated B cells (NF-κB), which is one of the main players in transcribing pro-inflammatory cytokines and, in turn, in increasing the expression of several proteins involved in the antioxidant response [47,57,58,59,60,61,62,63,64,65]. Among these proteins, Nrf2 has been demonstrated to play a crucial role in the activation of cytoprotective antioxidant genes against atmospheric pollutant-induced toxicity [66]. 

Similarly, exposure to low O_3_ concentrations for therapeutic purposes was found to act on Nrf2. Ozonated serum was able to activate Nrf2 in a dose dependent manner and to subsequently induce the expression of heme oxygenase 1 (HMOX1) and nicotinamide adenine dinucleotide phosphate (NAD(P)H) quinone oxidoreductase 1 (NQO1) in endothelial cells [67]. Systemic O_3_ treatment in healthy volunteers increased the levels of Nrf2 in peripheral blood mononuclear cells with consequent enhanced activity of superoxide dismutase and catalase [68]. The application of ozonated saline in an in vitro human keratinocyte model of wound healing proved the activation of Nrf2 pathways resulting in the increased expression of the HMOX1 gene [69]. Systemic O_3_ administration in rats with adenine-induced chronic kidney disease inhibited the NF-κB pathway and induced Nrf2 activation: this resulted in the up-regulation of antioxidant enzymes and the down-regulation of inflammatory cytokines in the kidney, with reduction of the renal insufficiency and tubulointerstitial injury [70]. Rectal insufflation of O_3_ to patients affected by multiple sclerosis increased Nrf2 phosphorylation and casein kinase 2 (CK2) expression in mononuclear cells, thus improving the activity of antioxidant enzymes and reducing the levels of pro-inflammatory cytokines [71]. The systemic administration of O_2_-O_3_ was beneficial in the rat model of streptozotocin-induced pancreatic damage by increasing the endogenous Nrf2 and glutathione-S-transferase (GST) in the pancreatic tissue [72]. O_3_ treatment in rats induced oxidative preconditioning by activating Nrf2, thus protecting the lung and myocardium from the ischemia-reperfusion injury, a major cause of cardiac and respiratory dysfunction during cardiovascular surgery, heart transplantation and cardiopulmonary bypass procedures [73,74]. O_2_-O_3_ therapy proved to be beneficial also for the treatment of diabetic complications and spinal pain by activating various antioxidant pathways involving hypoxia inducible factor-1α (HIF-1α), nuclear factor of activated T-cells (NFAT), activated protein-1 (AP-1) pathways, and Nrf2 [75].

Based on these observations, it was hypothesized that the antioxidant and anti-inflammatory effects of low O_3_ concentrations involve activation of Nrf2, which is thus considered as a key factor for the efficacy of O_2_-O_3_ treatments [76,77,78].

## 3. Biological Role of Nrf2 and Its Activation by Ozone Treatment

Nrf2 was first cloned in 1994 and identified as a member of the human cap‘n’collar basic-region (CNC) leucine zipper transcription factor family [79], which also includes nuclear factor erythroid 2 (NF-E2), nuclear factor erythroid 2-related factor 1 (Nrf1), nuclear factor erythroid 2-related factor 3 (Nrf3), BTB domain and CNC homolog 1 (BACH1), and BTB domain and CNC homolog 2 (BACH2). Nrf2 dimerizes with the small musculoaponeurotic fibrosarcoma (Maf) proteins to bind and mediate the transcription at a consensus sequence containing an AP-1 core motif [80]; this latter was identified on the promoter of the rat GST *Ya* subunit gene [81] and thereafter referred to as Antioxidant Responsive Element (ARE) [82]. Under basal conditions, Nrf2 is expressed at very low level, and is mainly sequestered in the cytoplasm by its specific inhibitor, Kelch-like ECH associated protein-1 (Keap-1) that also promotes its rapid degradation [83]. The effectiveness of this mechanism allows a rapid turnover of Nrf2, which displays a hemi-life of a few minutes. Under specific stimuli, Nrf2 dissociates from Keap1, translocates into the nucleus and transactivates ARE-driven genes [84]. By combining sub-nuclear tracking of Nrf2 localization and functional genetic engineering we have recently demonstrated that mild ozonisation increases the nuclear translocation of Nrf2 at transcriptionally active chromatin sites, inducing the Nrf2-mediated Keap1-dependent transcription of ARE-driven genes [85].

Twenty-five years of studies have contributed to identify more than two hundred ARE genes, whose expression is regulated by the Nrf2 transcriptional activity. These genes encode for proteins involved in a multitude of vital biological functions which include protein homeostasis, oxidative stress response, detoxication, DNA repair, proliferation, autophagy, mitochondrial biogenesis and function, inflammation, and the metabolism of lipids, carbohydrates and amino acids [86,87]. The impact of Nrf2 on transcriptome is amplified by the fact that Nrf2 may directly regulate or bidirectionally cross-talk with many other transcription factors such as Notch1 [88], the aryl hydrocarbon receptor (AhR) [89], the CCAAT/enhancer-binding protein (C/EBPB) [90], the peroxisome proliferator-activated receptor gamma (PPARγ) [91], the retinoid X receptor alpha (RXRA) and NF-κB. Finally, multiple lines of evidence have shown that Nrf2 activation is part of the retrograde response aimed at restoring mitochondrial function after stress insults, and that the impairment of Nrf2 functions is a hallmark of many mitochondrial-related disorders [92,93,94,95].

**Figure 1:**
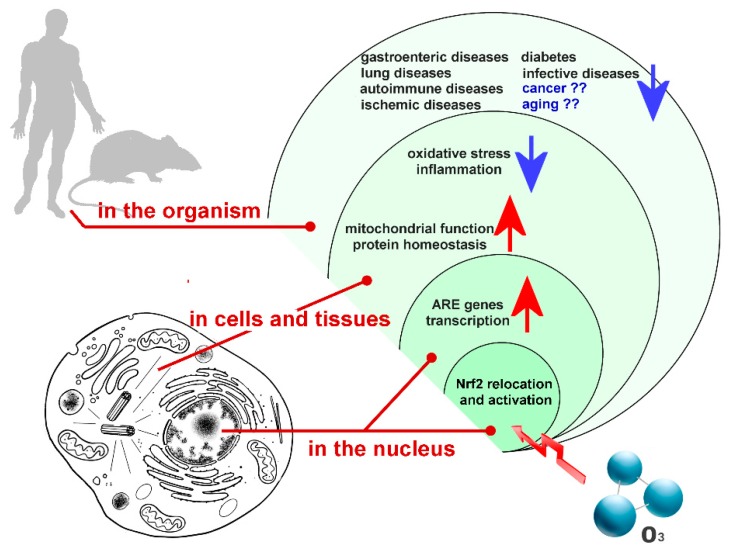
Main functional pathways depending on Nrf2 activation induced by low ozone concentrations. Arrows indicate up- (red) or down- (blue) regulation.

Some of the cell functional pathways that are dependent on Nrf2 activation (Figure 1) are summarized in the following paragraphs.

### 3.1. Nrf2 and Oxidative Stress

Oxidative stress is due to the accumulation of reactive oxygen species (ROS) (O_2_^−^, H_2_O_2_ and •OH), that are generated as by-products of either physiological or exogenous stress factors (such as, e.g., the ionizing radiations). Although ROS have been recognized in the last years as functionally significant signalling molecules for the modulation of the immune system, they have long been seen as harmful factors with detrimental effect on cell homeostasis. For example, ROS are strong inducers of DNA damage [96] that irreversibly compromises cell functions and might stimulate neoplastic transformation [97]. Nrf2 prevents oxidative stress through the transcription of antioxidant enzymes, such as the catalytic and modulatory subunits of glutamate cysteine ligase (GCL), glutathione peroxidases (GPX2 and GPX4), glutathione reductase (GSR), peroxiredoxins (PRDX1 and PRDX6), thioredoxin 1 and thioredoxin reductase 1 (TXN1 and TXNRD1), HMOX1, and biliverdin reductase (BVR) [87,98,99,100]. Accordingly, we demonstrated that mild ozonisation induces modulation of genes involved in the cell response to stress (HMOX1; excision repair cross-complementation group 4, ERCC4; cyclin-dependent kinase inhibitor 1A, CDKN1A) and in the transcription machinery (CTD small phosphatase 1, CTDSP1) [101].

### 3.2. Nrf2 and Proteostasis

Oxidative stress is one of the major drivers of protein misfolding as it induces protein oxidation. Misfolded or unfolded proteins accumulate as insoluble inclusions and aggregates in the cytoplasm and the cell nucleus, and are the hallmark of multiple aging-related neurodegenerative and metabolic disorders [102]. Nrf2 promotes the clearance of oxidized or otherwise damaged proteins through the two major pathways of protein degradation, i.e., the ubiquitin proteasome system and autophagy. Nrf2 directly targets the transcription of many proteasomal genes [103,104] and of the proteasome maturation protein (POMP) [105]. Nrf2 also supports autophagy either directly (by regulating the transcription of key autophagic genes, such as autophagy related ATG 5 and 7, unc-51-like autophagy activating kinase (ULK) 1 and 2, and the autophagy transporter ubiquitin-binding protein p62, sequestosome-1 (SQSTM1) [106], or indirectly through the activation of mammalian target of rapamycin (mTOR), a master regulator of protein synthesis and autophagy [107]. Consistently, O_3_ treatment proved to promote wound healing by increasing the migration of fibroblasts via the phosphoinositide 3-kinase (PI3K)/protein-kinase B (Akt)/mTOR signalling pathway [48], and to increase the level of autophagy in a chondrocyte model of osteoarthritis through the activation of AMP-activated protein kinase (AMPK)/mTOR [108]. 

### 3.3. Nrf2 and the Mitochondrial Function

The classic view of mitochondria as semi-independent organelles has recently been integrated by the evidence that, in response to environmental changes, they may coordinate inter-organelle signalling pathways (mitochondrial retrograde response) that ultimately instruct nuclear gene expression [109,110,111]. Maintenance of an efficient mitochondrial function is crucial to preserve cell homeostasis, as proven by the evidence that mitochondrial stimulation by mild-stress treatments (mitohormesis) [112] underlies the beneficial effects of life-extending interventions such as dietary restriction [113,114]. In contrast, mitochondrial dysfunction is ontologically linked (as either cause, con-cause or consequence) to aging and aging-related diseases [115,116]. Nrf2 has been shown to stimulate mitochondrial biogenesis through the activation of nuclear respiratory factor-1 (NRF-1) in cardiomyocytes [117,118]. Cellular respiration and ATP synthesis are impaired in conditions of Nrf2 deficiency and increased upon Keap1 loss of function [119,120]. Nrf2 is functionally linked through a positive feedback loop to Sqstm1/p62, which localizes to the mitochondria and enhances the mitochondrial transcription factor A (TFAM), a master regulator of mitochondrial biogenesis [121,122]. In addition, Nrf2 has been identified as a crucial mediator of the mitochondrial biogenesis induced by acetyl-carnitine (ALCAR), nitric oxide (NO) and resveratrol [123,124]. Accordingly, mild ozonisation was found to affect mitochondria by increasing the length of the mitochondrial cristae and the content of mitochondrial heat-shock protein 70 [125], while O_3_ treatment was proven to reduce mitochondrial damage in a rat heart following ischemia-reperfusion [73] as well as in a rat brain and cochlea following noise-induced hearing loss [126]. 

### 3.4. Nrf2 and Inflammation

Inflammatory pathways protect from exogenous harmful stimuli and, to some extent, from intrinsic dysregulation of cell proliferation that may lead to neoplastic growth. However, inflammation may also take place during aberrant self-reactivity in autoimmune diseases [127], while a low but chronic level of inflammation in the absence of real exogenous or endogenous threats is a common hallmark of aging (inflammaging) and aging-related diseases [128,129]. Nrf2 is able to modulate inflammation through multiple mechanisms, such as the regulation of redox homeostasis and the suppression of pro-inflammatory genes, either directly or through the interaction with NF-κB. Inflammation increases local and systemic ROS level while ROS enhance inflammation [130]. The Nrf2-mediated ROS-homeostatic control is able to break this vicious cycle. Nrf2 reduces inflammation by preventing the recruitment of RNA polymerase II to start gene transcription of pro-inflammatory cytokines IL-6 and IL1β [131]. In addition, Nrf2 tunes gene expression in inflammatory macrophages through a bidirectional crosstalk with NF-κB transcription factor, and regulates the transcription of Nrf2 itself [132] that, in turn, inhibits the transcriptional activity of NF-κB [133]. NF-κB has long been recognized as a point of convergence of inflammation and aging, and the array of NF-κB-regulated genes largely overlaps with the targets of transcriptional/epigenetic regulators such as sirtuins that have been demonstrated to promote lifespan extension [134,135,136]. This notion once again corroborates the ontological link between inflammation and aging and allows speculation that O_3_-induced activation of Nrf2 might be a potential tool to prevent aging and prolong lifespan. 

### 3.5. Nrf2 and Adipose Biology

Oxidative stress is a crucial factor in adipocyte differentiation, and ROS are known to promote adipogenesis via insulin-mediated signal transduction [137,138]. As an antioxidant regulator factor, Nrf2 is involved in the adipogenic differentiation of mesenchymal stem cells [90,139,140], together with HMOX-1 [141]. A recent study on 3T3-L1 cells suggested that Nrf2 affects adipocyte differentiation by modulating the expression of the fibroblast growth factor 21 (FGF21) through the PPARγ, a master transcription factor regulating inflammation as well as adipogenesis and insulin sensitization [142]. Consistently, treating the adipose tissue with low O_3_ concentrations proved to exert an adipogenic effect on human adipose-derived adult stem cells [143] in the absence of damage in differentiated adipocytes [144]; this allows foreseeing mild ozonisation as an adjuvant tool for tissue regeneration and engineering. 

### 3.6. Nrf2 and Cancer

Unlike the vast majority of diseases that have an often unique pathogenic cause, cancer can be defined as multitude of possible pathologic states sharing the capability to subvert and redirect the regeneration and differentiation potential of cells and tissues toward abnormal limitless proliferation and growth. The Nrf2-mediated regulation of the biological processes described above has proven to exert beneficial effects in preventing, ameliorating or curing a multitude of diseases, but might turn detrimental in a cancer-related context. Nrf2 preserves cells from DNA damage, and therefore might help preventing the primary trigger of neoplastic transformation. However, hyperactivation of Nrf2 has been shown to support tumour progression by multiple ways [145,146]: for example, it may help incipient tumour cells to overcome oxidative stress that represents a barrier against neoplastic transformation and cancer initiation [147]. Also, Nrf2 hyperactivation supports aberrant cell proliferation by both inducing the metabolic switch towards anabolic pathways [148] and modulating mRNA translation [149]. Moreover, Nrf2 may promote tumour angiogenesis [150]. Finally, the potent cytoprotective effect of Nrf2 activation may confer drug resistance to cancer cells [151]. Thus, the possible tumour-promoting effect of Nrf2 hyperactivation still remains a crucial issue, since oncological patients are frequently administered O_3_ therapy due to its efficacy in reducing some adverse side-effects of the anti-cancer treatments [46,152,153].

## 4. Conclusions

As a whole, the direct and indirect molecular targets of Nrf2 delineates a complex network of biological processes that preserve cell homeostasis and promote cell reparative programs following chemical, physical, or biological stress [154]. The complexity of the Nrf2 functional network does not allow drawing an exhaustive molecular model that might explain the beneficial effects of O_3_ in preventing or ameliorating diseases. Nrf2 activation exerts positive effects especially on diseases that have oxidative stress and inflammation as primary etiopathological events [155,156]. Therefore, it may be hypothesized that the therapeutic potential of Nrf2 activation as a consequence of mild ozonisation relies on the capability of Nrf2 to maintain redox homeostasis: this would prevent DNA damage, preserve proteostasis, and improve mitochondrial function while suppressing acute and chronic inflammation.

## Abbreviations

Nrf2	nuclear factor erythroid 2-related factor 2
NF-κB	nuclear factor kappa-light-chain-enhancer of activated B cells
HMOX1	heme oxygenase 1
CK2	casein kinase 2
NAD(P)H	nicotinamide adenine dinucleotide phosphate
NQO1	nicotinamide adenine dinucleotide phosphate quinone oxidoreductase 1
GST	glutathione-S-transferase
HIF-1α	hypoxia inducible factor-1α
NFAT	nuclear factor of activated T-cells
AP-1	activated protein-1
CNC	cap‘n’collar basic-region
NF-E2	nuclear factor erythroid 2
Nrf1	nuclear factor erythroid 2-related factor 1
Nrf3	nuclear factor erythroid 2-related factor 3
BACH1	BTB domain and CNC homolog 1
BACH2	BTB domain and CNC homolog 2
Maf	musculoaponeurotic fibrosarcoma
ARE	Antioxidant Responsive Element
Keap-1	Kelch-like ECH associated protein-1
AhR	aryl hydrocarbon receptor
C/EBPB	CCAAT/enhancer-binding protein
PPARγ	peroxisome proliferator-activated receptor gamma
RXRA	retinoid X receptor alpha
ROS	reactive oxygen species
GCL	glutamate cysteine ligase
GPX	glutathione peroxidase
GSR	glutathione reductase
PRDX	peroxiredoxin
TXN1	thioredoxin 1
TXNRD1	thioredoxin reductase 1
BVR	biliverdin reductase
ERCC4	excision repair cross-complementation group 4
CDKN1A	cyclin-dependent kinase inhibitor 1A
CTDSP1	CTD small phosphatase 1
POMP	proteasome maturation protein
ATG	autophagy related
ULK	unc-51-like autophagy activating kinase
SQSTM1	sequestosome-1
mTOR	mammalian target of rapamycin
PI3K	phosphoinositide 3-kinase
Akt	protein-kinase B
AMPK	adenosine monophosphate-activated protein kinase
NRF-1	nuclear respiratory factor-1
TFAM	mitochondrial transcription factor A
ALCAR	acetyl-carnitine
NO	nitric oxide
FGF21	fibroblast growth factor 21

## References

[1] Fuks K.B., Woodby B., Valacchi G. (2019). Skin damage by tropospheric ozone. Hautarzt.

[2] Zu K., Shi L., Prueitt R.L., Liu X., Goodman J.E. (2018). Critical review of long-term ozone exposure and asthma development. Inhal. Toxicol..

[3] Snow S.J., Henriquez A.R., Costa D.L., Kodavanti U.P. (2018). Neuroendocrine Regulation of Air Pollution Health Effects: Emerging Insights. Toxicol. Sci..

[4] Jung S.J., Mehta J.S., Tong L. (2018). Effects of environment pollution on the ocular surface. Ocul. Surf..

[5] Croze M.L., Zimmer L. (2018). Ozone Atmospheric Pollution and Alzheimer’s Disease: From Epidemiological Facts to Molecular Mechanisms. J. Alzheimers Dis..

[6] Leduc C., Daneault C., Pelletier É., Cloutier J.-N., Epiney M., Lanouette R. (2018). Bleaching efficiency of softwood thermomechanical pulps treated with ozone. Appita. J..

[7] Alresheedi M.T., Basu O.D., Barbeau B. (2019). Chemical cleaning of ceramic ultrafiltration membranes—Ozone versus conventional cleaning chemicals. Chemosphere.

[8] Englezos V., Rantsiou K., Cravero F., Torchio F., Giacosa S., Río Segade S., Gai G., Dogliani E., Gerbi V., Cocolin L. (2019). Minimizing the environmental impact of cleaning in winemaking industry by using ozone for cleaning-in-place (CIP) of wine bottling machine. J. Clean. Prod..

[9] Jin X., Wang W., Wang S., Jin P., Wang X.C., Zhang W., An W., Wang Y. (2019). Application of a hybrid gravity-driven membrane filtration and dissolved ozone flotation (MDOF) process for wastewater reclamation and membrane fouling mitigation. J. Environ. Sci.-China.

[10] Zanacic E., Stavrinides J., McMartin D.W. (2016). Field-analysis of potable water quality and ozone efficiency in ozone-assisted biological filtration systems for surface water treatment. Water Res..

[11] Martinelli M., Giovannangeli F., Rotunno S., Trombetta C.M., Montomoli E. (2017). Water and air ozone treatment as an alternative sanitizing technology. J. Prev. Med. Hyg..

[12] Brodowska A.J., Nowak A., Śmigielski K. (2018). Ozone in the food industry: Principles of ozone treatment, mechanisms of action, and applications: An overview. Crit. Rev. Food Sci. Nutr..

[13] Cisterna B., Boschi F., Croce A.C., Podda R., Zanzoni S., Degl’Innocenti D., Bernardi P., Costanzo M., Marzola P., Covi V. (2018). Ozone Treatment of Grapes During Withering for Amarone Wine: A Multimodal Imaging and Spectroscopic Analysis. Microsc. Microanal..

[14] Viebahn-Hansler R. (2007). The Use of Ozone in Medicine.

[15] Viebahn-Hansler R., Leon Fernandez O.S., Fahmy Z. (2012). Ozone in medicine: The low dose ozone concept—Guidelines and treatment strategies. Ozone Sci. Engeneering.

[16] Goldman M. (1996). Cancer risk of low-level exposure. Science.

[17] Bocci V.A., Zanardi I., Travagli V. (2011). Ozone acting on human blood yields a hormetic dose-response relationship. J. Transl. Med..

[18] Bocci V. (2005). General mechanism of action of ozone therapy and mechanism in pain treatment. Rev. Soc. Esp. Dolor..

[19] Borrelli E. (2011). Mechanism of action of oxygen ozone therapy in the treatment of disc herniation and low back pain. Acta Neurochir. Suppl..

[20] Magalhaes F.N., Dotta L., Sasse A., Teixera M.J., Fonoff E.T. (2012). Ozone therapy as a treatment for low back pain secondary to herniated disc: A systematic review and meta-analysis of randomized controlled trials. Pain Physician.

[21] Muto M., Giurazza F., Silva R.P., Guarnieri G. (2016). Rational approach, technique and selection criteria treating lumbar disk herniations by oxygen-ozone therapy. Interv. Neuroradiol..

[22] Lopes de Jesus C.C., Dos Santos F.C., de Jesus L.M.O.B., Monteiro I., Sant’Ana M.S.S.C., Trevisani V.F.M. (2017). Comparison between intra-articular ozone and placebo in the treatment of knee osteoarthritis: A randomized, double-blinded, placebo-controlled study. PLoS ONE.

[23] Schulz S. (1986). The role of ozone/oxygen in clindamycin-associated enterocolitis in the Djungarian hamster (Phodopus sungorus sungorus). Lab Anim..

[24] Carpendale M.T., Freeberg J., Griffiss J.M. (1993). Does ozone alleviate AIDS diarrhea?. J. Clin. Gastroenterol..

[25] Zamora Rodríguez Z.B., González Alvarez R., Guanche D., Merino N., Hernández Rosales F., Menéndez Cepero S., Alonso González Y., Schulz S. (2007). Antioxidant mechanism is involved in the gastroprotective effects of ozonized sunflower oil in ethanol-induced ulcers in rats. Mediat. Inflamm..

[26] Altinel O., Demirbas S., Cakir E., Yaman H., Ozerhan I.H., Duran E., Cayci T., Akgul E.O., Ersoz N., Uysal B. (2011). Comparison of hyperbaric oxygen and medical ozone therapies in a rat model of experimental distal colitis. Scand. J. Clin. Lab. Invest..

[27] Aslaner A., Çakır T., Tekeli S., Avcı S., Doğan U., Tekeli F., Soylu H., Akyüz C., Koç S., Üstünel İ. (2016). Medical ozone treatment ameliorates the acute distal colitis in rat. Acta Cir. Bras..

[28] Hernández Rosales F.A., Calunga Fernández J.L., Turrent Figueras J., Menéndez Cepero S., Montenegro Perdomo A. (2005). Ozone therapy effects on biomarkers and lung function in asthma. Arch. Med. Res..

[29] Kaldirim U., Uysal B., Yuksel R., Macit E., Eyi Y.E., Toygar M., Tuncer S.K., Ardic S., Arziman I., Aydin I. (2014). Ozone therapy ameliorates paraquat-induced lung injury in rats. Exp. Biol. Med. (Maywood).

[30] Kucukgul A., Erdogan S., Gonenci R., Ozan G. (2016). Beneficial effects of nontoxic ozone on H. Biochem. Cell Biol..

[31] Santana-Rodríguez N., Llontop P., Clavo B., Fiuza-Pérez M.D., Zerecero K., Ayub A., Alshehri K., Yordi N.A., Re L., Raad W. (2017). Ozone Therapy Protects Against Rejection in a Lung Transplantation Model: A New Treatment?. Ann. Thorac. Surg..

[32] Martínez-Sánchez G., Al-Dalain S.M., Menéndez S., Re L., Giuliani A., Candelario-Jalil E., Alvarez H., Fernández-Montequín J.I., León O.S. (2005). Therapeutic efficacy of ozone in patients with diabetic foot. Eur. J. Pharm..

[33] Alpan A.L., Toker H., Ozer H. (2016). Ozone Therapy Enhances Osseous Healing in Rats With Diabetes With Calvarial Defects: A Morphometric and Immunohistochemical Study. J. Periodontol..

[34] Güçlü A., Erken H.A., Erken G., Dodurga Y., Yay A., Özçoban Ö., Şimşek H., Akçılar A., Koçak F.E. (2016). The effects of ozone therapy on caspase pathways, TNF-α, and HIF-1α in diabetic nephropathy. Int. Urol. Nephrol..

[35] Rosul M.V., Patskan B.M. (2016). Ozone therapy effectiveness in patients with ulcerous lesions due to diabetes mellitus. Wiad. Lek..

[36] Xie T.Y., Yan W., Lou J., Chen X.Y. (2016). Effect of ozone on vascular endothelial growth factor (VEGF) and related inflammatory cytokines in rats with diabetic retinopathy. Genet. Mol. Res..

[37] Izadi M., Bozorgi M., Hosseine M.S., Khalili N., Jonaidi-Jafari N. (2018). Health-related quality of life in patients with chronic wounds before and after treatment with medical ozone. Medicine (Baltimore).

[38] León O.S., Menéndez S., Merino N., Castillo R., Sam S., Pérez L., Cruz E., Bocci V. (1998). Ozone oxidative preconditioning: A protection against cellular damage by free radicals. Mediat. Inflamm..

[39] Ajamieh H.H., Menéndez S., Martínez-Sánchez G., Candelario-Jalil E., Re L., Giuliani A., Fernández O.S. (2004). Effects of ozone oxidative preconditioning on nitric oxide generation and cellular redox balance in a rat model of hepatic ischaemia-reperfusion. Liver Int..

[40] Ajamieh H.H., Berlanga J., Merino N., Sánchez G.M., Carmona A.M., Cepero S.M., Giuliani A., Re L., León O.S. (2005). Role of protein synthesis in the protection conferred by ozone-oxidative-preconditioning in hepatic ischaemia/reperfusion. Transpl. Int..

[41] Haj B., Sukhotnik I., Shaoul R., Pollak Y., Coran A.G., Bitterman A., Matter I. (2014). Effect of ozone on intestinal recovery following intestinal ischemia-reperfusion injury in a rat. Pediatr. Surg. Int..

[42] Isik A., Peker K., Gursul C., Sayar I., Firat D., Yilmaz I., Demiryilmaz I. (2015). The effect of ozone and naringin on intestinal ischemia/reperfusion injury in an experimental model. Int. J. Surg..

[43] Sancak E.B., Turkön H., Çukur S., Erimsah S., Akbas A., Gulpinar M.T., Toman H., Sahin H., Uzun M. (2016). Major Ozonated Autohemotherapy Preconditioning Ameliorates Kidney Ischemia-Reperfusion Injury. Inflammation.

[44] Sweet F., Kao M.S., Lee S.C., Hagar W.L., Sweet W.E. (1980). Ozone selectively inhibits growth of human cancer cells. Science.

[45] Tirelli U., Cirrito C., Pavanello M., Del Pup L., Lleshi A., Berretta M. (2018). Oxygen-ozone therapy as support and palliative therapy in 50 cancer patients with fatigue—A short report. Eur. Rev. Med. Pharm. Sci..

[46] Luongo M., Brigida A.L., Mascolo L., Gaudino G. (2017). Possible Therapeutic Effects of Ozone Mixture on Hypoxia in Tumor Development. Anticancer Res..

[47] Wells K.H., Latino J., Gavalchin J., Poiesz B.J. (1991). Inactivation of human immunodeficiency virus type 1 by ozone in vitro. Blood.

[48] Xiao W., Tang H., Wu M., Liao Y., Li K., Li L., Xu X. (2017). Ozone oil promotes wound healing by increasing the migration of fibroblasts via PI3K/Akt/mTOR signaling pathway. Biosci. Rep..

[49] Song M., Zeng Q., Xiang Y., Gao L., Huang J., Wu K., Lu J. (2018). The antibacterial effect of topical ozone on the treatment of MRSA skin infection. Mol. Med. Rep..

[50] Amin L.E. (2018). Biological assessment of ozone therapy on experimental oral candidiasis in immunosuppressed rats. Biochem. Biophys. Rep..

[51] Azarpazhooh A., Limeback H. (2008). The application of ozone in dentistry: A systematic review of literature. J. Dent..

[52] Gupta G., Mansi B. (2012). Ozone therapy in periodontics. J. Med. Life.

[53] Srikanth A., Sathish M., Sri Harsha A.V. (2013). Application of ozone in the treatment of periodontal disease. J. Pharm. Bioallied. Sci..

[54] Khatri I., Moger G., Kumar N.A. (2015). Evaluation of effect of topical ozone therapy on salivary Candidal carriage in oral candidiasis. Indian. J. Dent. Res..

[55] Al-Omiri M.K., Abul Hassan R.S., AlZarea B.K., Lynch E. (2016). Comparison of dental bleaching effects of ozone and hydrogen peroxide: An ex vivo study. Am. J. Dent..

[56] Isler S.C., Unsal B., Soysal F., Ozcan G., Peker E., Karaca I.R. (2018). The effects of ozone therapy as an adjunct to the surgical treatment of peri-implantitis. J. Periodontal. Implant. Sci..

[57] Kim M.Y., Song K.S., Park G.H., Chang S.H., Kim H.W., Park J.H., Jin H., Eu K.J., Cho H.S., Kang G. (2004). B6C3F1 mice exposed to ozone with 4-(N-methyl-N-nitrosamino)-1-(3-pyridyl)-1-butanone and/or dibutyl phthalate showed toxicities through alterations of NF-kappaB, AP-1, Nrf2, and osteopontin. J. Vet. Sci..

[58] Cho H.Y., Gladwell W., Yamamoto M., Kleeberger S.R. (2013). Exacerbated airway toxicity of environmental oxidant ozone in mice deficient in Nrf2. Oxid. Med. Cell. Longev..

[59] Wiegman C.H., Li F., Clarke C.J., Jazrawi E., Kirkham P., Barnes P.J., Adcock I.M., Chung K.F. (2014). A comprehensive analysis of oxidative stress in the ozone-induced lung inflammation mouse model. Clin. Sci. (Lond.).

[60] Ward W.O., Ledbetter A.D., Schladweiler M.C., Kodavanti U.P. (2015). Lung transcriptional profiling: Insights into the mechanisms of ozone-induced pulmonary injury in Wistar Kyoto rats. Inhal. Toxicol..

[61] Yan Z., Jin Y., An Z., Liu Y., Samet J.M., Wu W. (2016). Inflammatory cell signaling following exposures to particulate matter and ozone. Biochim. Biophys. Acta.

[62] Zhong J., Allen K., Rao X., Ying Z., Braunstein Z., Kankanala S.R., Xia C., Wang X., Bramble L.A., Wagner J.G. (2016). Repeated ozone exposure exacerbates insulin resistance and activates innate immune response in genetically susceptible mice. Inhal. Toxicol..

[63] Bromberg P.A. (2016). Mechanisms of the acute effects of inhaled ozone in humans. Biochim. Biophys. Acta.

[64] Traboulsi H., Guerrina N., Iu M., Maysinger D., Ariya P., Baglole C.J. (2017). Inhaled Pollutants: The Molecular Scene behind Respiratory and Systemic Diseases Associated with Ultrafine Particulate Matter. Int. J. Mol. Sci..

[65] Kim B.G., Lee P.H., Lee S.H., Park C.S., Jang A.S. (2018). Impact of ozone on claudins and tight junctions in the lungs. Environ. Toxicol..

[66] Rubio V., Valverde M., Rojas E. (2010). Effects of atmospheric pollutants on the Nrf2 survival pathway. Environ. Sci. Pollut. Res. Int..

[67] Pecorelli A., Bocci V., Acquaviva A., Belmonte G., Gardi C., Virgili F., Ciccoli L., Valacchi G. (2013). NRF2 activation is involved in ozonated human serum upregulation of HO-1 in endothelial cells. Toxicol. Appl. Pharm..

[68] Re L., Martínez-Sánchez G., Bordicchia M., Malcangi G., Pocognoli A., Morales-Segura M.A., Rothchild J., Rojas A. (2014). Is ozone pre-conditioning effect linked to Nrf2/EpRE activation pathway in vivo? A preliminary result. Eur. J. Pharm..

[69] Valacchi G., Sticozzi C., Zanardi I., Belmonte G., Cervellati F., Bocci V., Travagli V. (2016). Ozone mediators effect on “in vitro” scratch wound closure. Free Radic. Res..

[70] Yu G., Liu X., Chen Z., Chen H., Wang L., Wang Z., Qiu T., Weng X. (2016). Ozone therapy could attenuate tubulointerstitial injury in adenine-induced CKD rats by mediating Nrf2 and NF-κB. Iran. J. Basic. Med. Sci..

[71] Delgado-Roche L., Riera-Romo M., Mesta F., Hernández-Matos Y., Barrios J.M., Martínez-Sánchez G., Al-Dalaien S.M. (2017). Medical ozone promotes Nrf2 phosphorylation reducing oxidative stress and pro-inflammatory cytokines in multiple sclerosis patients. Eur. J. Pharm..

[72] Siniscalco D., Trotta M.C., Brigida A.L., Maisto R., Luongo M., Ferraraccio F., D’Amico M., Di Filippo C. (2018). Intraperitoneal Administration of Oxygen/Ozone to Rats Reduces the Pancreatic Damage Induced by Streptozotocin. Biology.

[73] Meng W., Xu Y., Li D., Zhu E., Deng L., Liu Z., Zhang G., Liu H. (2017). Ozone protects rat heart against ischemia-reperfusion injury: A role for oxidative preconditioning in attenuating mitochondrial injury. Biomed. Pharm..

[74] Wang Z., Zhang A., Meng W., Wang T., Li D., Liu Z., Liu H. (2018). Ozone protects the rat lung from ischemia-reperfusion injury by attenuating NLRP3-mediated inflammation, enhancing Nrf2 antioxidant activity and inhibiting apoptosis. Eur. J. Pharm..

[75] Braidy N., Izadi M., Sureda A., Jonaidi-Jafari N., Banki A., Nabavi S.F., Nabavi S.M. (2018). Therapeutic relevance of ozone therapy in degenerative diseases: Focus on diabetes and spinal pain. J. Cell. Physiol..

[76] Sagai M., Bocci V. (2011). Mechanisms of Action Involved in Ozone Therapy: Is healing induced via a mild oxidative stress?. Med. Gas. Res..

[77] Bocci V. (2012). How a calculated oxidative stress can yield multiple therapeutic effects. Free Radic. Res..

[78] Bocci V., Valacchi G. (2015). Nrf2 activation as target to implement therapeutic treatments. Front. Chem..

[79] Moi P., Chan K., Asunis I., Cao A., Kan Y.W. (1994). Isolation of NF-E2-related factor 2 (Nrf2), a NF-E2-like basic leucine zipper transcriptional activator that binds to the tandem NF-E2/AP1 repeat of the beta-globin locus control region. Proc. Natl. Acad. Sci. USA.

[80] Itoh K., Chiba T., Takahashi S., Ishii T., Igarashi K., Katoh Y., Oyake T., Hayashi N., Satoh K., Hatayama I. (1997). An Nrf2/small Maf heterodimer mediates the induction of phase II detoxifying enzyme genes through antioxidant response elements. Biochem. Biophys. Res. Commun..

[81] Rushmore T.H., Pickett C.B. (1990). Transcriptional regulation of the rat glutathione S-transferase Ya subunit gene. Characterization of a xenobiotic-responsive element controlling inducible expression by phenolic antioxidants. J. Biol. Chem..

[82] Rushmore T.H., Morton M.R., Pickett C.B. (1991). The antioxidant responsive element. Activation by oxidative stress and identification of the DNA consensus sequence required for functional activity. J. Biol. Chem..

[83] Itoh K., Wakabayashi N., Katoh Y., Ishii T., Igarashi K., Engel J.D., Yamamoto M. (1999). Keap1 represses nuclear activation of antioxidant responsive elements by Nrf2 through binding to the amino-terminal Neh2 domain. Genes Dev..

[84] Furukawa M., Xiong Y. (2005). BTB protein Keap1 targets antioxidant transcription factor Nrf2 for ubiquitination by the Cullin 3-Roc1 ligase. Mol. Cell. Biol..

[85] Galiè M., Costanzo M., Nodari A., Boschi F., Calderan L., Mannucci S., Covi V., Tabaracci G., Malatesta M. (2018). Mild ozonisation activates antioxidant cell response by the Keap1/Nrf2 dependent pathway. Free Radic. Biol. Med..

[86] Hybertson B.M., Gao B., Bose S.K., McCord J.M. (2011). Oxidative stress in health and disease: The therapeutic potential of Nrf2 activation. Mol. Aspects Med..

[87] Hayes J.D., Dinkova-Kostova A.T. (2014). The Nrf2 regulatory network provides an interface between redox and intermediary metabolism. Trends Biochem. Sci..

[88] Wakabayashi N., Shin S., Slocum S.L., Agoston E.S., Wakabayashi J., Kwak M.K., Misra V., Biswal S., Yamamoto M., Kensler T.W. (2010). Regulation of notch1 signaling by nrf2: Implications for tissue regeneration. Sci. Signal..

[89] Shin S., Wakabayashi N., Misra V., Biswal S., Lee G.H., Agoston E.S., Yamamoto M., Kensler T.W. (2007). NRF2 modulates aryl hydrocarbon receptor signaling: Influence on adipogenesis. Mol. Cell. Biol..

[90] Hou Y., Xue P., Bai Y., Liu D., Woods C.G., Yarborough K., Fu J., Zhang Q., Sun G., Collins S. (2012). Nuclear factor erythroid-derived factor 2-related factor 2 regulates transcription of CCAAT/enhancer-binding protein β during adipogenesis. Free Radic. Biol. Med..

[91] Chorley B.N., Campbell M.R., Wang X., Karaca M., Sambandan D., Bangura F., Xue P., Pi J., Kleeberger S.R., Bell D.A. (2012). Identification of novel NRF2-regulated genes by ChIP-Seq: Influence on retinoid X receptor alpha. Nucleic Acids Res..

[92] Pulliam D.A., Deepa S.S., Liu Y., Hill S., Lin A.L., Bhattacharya A., Shi Y., Sloane L., Viscomi C., Zeviani M. (2014). Complex IV-deficient Surf1(-/-) mice initiate mitochondrial stress responses. Biochem. J..

[93] Paupe V., Dassa E.P., Goncalves S., Auchère F., Lönn M., Holmgren A., Rustin P. (2009). Impaired nuclear Nrf2 translocation undermines the oxidative stress response in Friedreich ataxia. PLoS ONE.

[94] Shan Y., Schoenfeld R.A., Hayashi G., Napoli E., Akiyama T., Iodi Carstens M., Carstens E.E., Pook M.A., Cortopassi G.A. (2013). Frataxin deficiency leads to defects in expression of antioxidants and Nrf2 expression in dorsal root ganglia of the Friedreich’s ataxia YG8R mouse model. Antioxid. Redox. Signal..

[95] D’Oria V., Petrini S., Travaglini L., Priori C., Piermarini E., Petrillo S., Carletti B., Bertini E., Piemonte F. (2013). Frataxin deficiency leads to reduced expression and impaired translocation of NF-E2-related factor (Nrf2) in cultured motor neurons. Int. J. Mol. Sci..

[96] Commoner B., Townsend J., Pake G.E. (1954). Free radicals in biological materials. Nature.

[97] Kruk J., Aboul-Enein H.Y., Kładna A., Bowser J.E. (2019). Oxidative stress in biological systems and its relation with pathophysiological functions: The effect of physical activity on cellular redox homeostasis. Free Radic. Res..

[98] Tanito M., Agbaga M.P., Anderson R.E. (2007). Upregulation of thioredoxin system via Nrf2-antioxidant responsive element pathway in adaptive-retinal neuroprotection in vivo and in vitro. Free Radic. Biol. Med..

[99] MacLeod A.K., McMahon M., Plummer S.M., Higgins L.G., Penning T.M., Igarashi K., Hayes J.D. (2009). Characterization of the cancer chemopreventive NRF2-dependent gene battery in human keratinocytes: Demonstration that the KEAP1-NRF2 pathway, and not the BACH1-NRF2 pathway, controls cytoprotection against electrophiles as well as redox-cycling compounds. Carcinogenesis.

[100] Agyeman A.S., Chaerkady R., Shaw P.G., Davidson N.E., Visvanathan K., Pandey A., Kensler T.W. (2012). Transcriptomic and proteomic profiling of KEAP1 disrupted and sulforaphane-treated human breast epithelial cells reveals common expression profiles. Breast Cancer Res. Treat..

[101] Scassellati C., Costanzo M., Cisterna B., Nodari A., Galiè M., Cattaneo A., Covi V., Tabaracci G., Bonvicini C., Malatesta M. (2017). Effects of mild ozonisation on gene expression and nuclear domains organization in vitro. Toxicol. Vitr..

[102] Knowles T.P., Vendruscolo M., Dobson C.M. (2014). The amyloid state and its association with protein misfolding diseases. Nat. Rev. Mol. Cell. Biol..

[103] Kwak M.K., Wakabayashi N., Greenlaw J.L., Yamamoto M., Kensler T.W. (2003). Antioxidants enhance mammalian proteasome expression through the Keap1-Nrf2 signaling pathway. Mol. Cell. Biol..

[104] Kapeta S., Chondrogianni N., Gonos E.S. (2010). Nuclear erythroid factor 2-mediated proteasome activation delays senescence in human fibroblasts. J. Biol Chem.

[105] Jang J., Wang Y., Kim H.S., Lalli M.A., Kosik K.S. (2014). Nrf2, a regulator of the proteasome, controls self-renewal and pluripotency in human embryonic stem cells. Stem Cells.

[106] Pajares M., Jiménez-Moreno N., García-Yagüe Á., Escoll M., de Ceballos M.L., Van Leuven F., Rábano A., Yamamoto M., Rojo A.I., Cuadrado A. (2016). Transcription factor NFE2L2/NRF2 is a regulator of macroautophagy genes. Autophagy.

[107] Bendavit G., Aboulkassim T., Hilmi K., Shah S., Batist G. (2016). Nrf2 Transcription Factor Can Directly Regulate mTOR: Linking Cytoprotective Gene Expression to a Major Metabolic Regulator That Generates Redox Activity. J. Biol. Chem..

[108] Zhao X., Li Y., Lin X., Wang J., Xie J., Sun T., Fu Z. (2018). Ozone induces autophagy in rat chondrocytes stimulated with IL-1β through the AMPK/mTOR signaling pathway. J. Pain Res..

[109] Chandel N.S. (2015). Evolution of Mitochondria as Signaling Organelles. Cell Metab..

[110] Cardamone M.D., Tanasa B., Cederquist C.T., Huang J., Mahdaviani K., Li W., Rosenfeld M.G., Liesa M., Perissi V. (2018). Mitochondrial Retrograde Signaling in Mammals Is Mediated by the Transcriptional Cofactor GPS2 via Direct Mitochondria-to-Nucleus Translocation. Mol. Cell.

[111] Quirós P.M., Prado M.A., Zamboni N., D’Amico D., Williams R.W., Finley D., Gygi S.P., Auwerx J. (2017). Multi-omics analysis identifies ATF4 as a key regulator of the mitochondrial stress response in mammals. J. Cell Biol..

[112] Yun J., Finkel T. (2014). Mitohormesis. Cell Metab..

[113] Zid B.M., Rogers A.N., Katewa S.D., Vargas M.A., Kolipinski M.C., Lu T.A., Benzer S., Kapahi P. (2009). 4E-BP extends lifespan upon dietary restriction by enhancing mitochondrial activity in Drosophila. Cell.

[114] Linford N.J., Beyer R.P., Gollahon K., Krajcik R.A., Malloy V.L., Demas V., Burmer G.C., Rabinovitch P.S. (2007). Transcriptional response to aging and caloric restriction in heart and adipose tissue. Aging Cell.

[115] Sun N., Youle R.J., Finkel T. (2016). The Mitochondrial Basis of Aging. Mol. Cell.

[116] Wallace D.C. (2013). A mitochondrial bioenergetic etiology of disease. J. Clin. Investig..

[117] Piantadosi C.A., Carraway M.S., Babiker A., Suliman H.B. (2008). Heme oxygenase-1 regulates cardiac mitochondrial biogenesis via Nrf2-mediated transcriptional control of nuclear respiratory factor-1. Circ. Res..

[118] Merry T.L., Ristow M. (2016). Nuclear factor erythroid-derived 2-like 2 (NFE2L2, Nrf2) mediates exercise-induced mitochondrial biogenesis and the anti-oxidant response in mice. J. Physiol..

[119] Holmström K.M., Baird L., Zhang Y., Hargreaves I., Chalasani A., Land J.M., Stanyer L., Yamamoto M., Dinkova-Kostova A.T., Abramov A.Y. (2013). Nrf2 impacts cellular bioenergetics by controlling substrate availability for mitochondrial respiration. Biol. Open.

[120] Kovac S., Angelova P.R., Holmström K.M., Zhang Y., Dinkova-Kostova A.T., Abramov A.Y. (2015). Nrf2 regulates ROS production by mitochondria and NADPH oxidase. Biochim. Biophys. Acta.

[121] Kosaka K., Mimura J., Itoh K., Satoh T., Shimojo Y., Kitajima C., Maruyama A., Yamamoto M., Shirasawa T. (2010). Role of Nrf2 and p62/ZIP in the neurite outgrowth by carnosic acid in PC12h cells. J. Biochem..

[122] Seibenhener M.L., Du Y., Diaz-Meco M.T., Moscat J., Wooten M.C., Wooten M.W. (2013). A role for sequestosome 1/p62 in mitochondrial dynamics, import and genome integrity. Biochim. Biophys. Acta.

[123] Hota K.B., Hota S.K., Chaurasia O.P., Singh S.B. (2012). Acetyl-L-carnitine-mediated neuroprotection during hypoxia is attributed to ERK1/2-Nrf2-regulated mitochondrial biosynthesis. Hippocampus.

[124] Kim S.K., Joe Y., Zheng M., Kim H.J., Yu J.K., Cho G.J., Chang K.C., Kim H.K., Han J., Ryter S.W. (2014). Resveratrol induces hepatic mitochondrial biogenesis through the sequential activation of nitric oxide and carbon monoxide production. Antioxid. Redox Signal..

[125] Costanzo M., Cisterna B., Vella A., Cestari T., Covi V., Tabaracci G., Malatesta M. (2015). Low ozone concentrations stimulate cytoskeletal organization, mitochondrial activity and nuclear transcription. Eur. J. Histochem..

[126] Liu X., Wang S., You Y., Meng M., Zheng Z., Dong M., Lin J., Zhao Q., Zhang C., Yuan X. (2015). Brown Adipose Tissue Transplantation Reverses Obesity in Ob/Ob Mice. Endocrinology.

[127] Theofilopoulos A.N., Kono D.H., Baccala R. (2017). The multiple pathways to autoimmunity. Nat. Immunol..

[128] Franceschi C., Campisi J. (2014). Chronic inflammation (inflammaging) and its potential contribution to age-associated diseases. J. Gerontol. A Biol. Sci. Med. Sci..

[129] Franceschi C., Garagnani P., Morsiani C., Conte M., Santoro A., Grignolio A., Monti D., Capri M., Salvioli S. (2018). The Continuum of Aging and Age-Related Diseases: Common Mechanisms but Different Rates. Front. Med. (Lausanne).

[130] Joseph J., Ametepe E.S., Haribabu N., Agbayani G., Krishnan L., Blais A., Sad S. (2016). Inhibition of ROS and upregulation of inflammatory cytokines by FoxO3a promotes survival against Salmonella typhimurium. Nat. Commun..

[131] Kobayashi E.H., Suzuki T., Funayama R., Nagashima T., Hayashi M., Sekine H., Tanaka N., Moriguchi T., Motohashi H., Nakayama K. (2016). Nrf2 suppresses macrophage inflammatory response by blocking proinflammatory cytokine transcription. Nat. Commun..

[132] Rushworth S.A., Zaitseva L., Murray M.Y., Shah N.M., Bowles K.M., MacEwan D.J. (2012). The high Nrf2 expression in human acute myeloid leukemia is driven by NF-κB and underlies its chemo-resistance. Blood.

[133] Thimmulappa R.K., Lee H., Rangasamy T., Reddy S.P., Yamamoto M., Kensler T.W., Biswal S. (2006). Nrf2 is a critical regulator of the innate immune response and survival during experimental sepsis. J. Clin. Investig..

[134] Kawahara T.L., Michishita E., Adler A.S., Damian M., Berber E., Lin M., McCord R.A., Ongaigui K.C., Boxer L.D., Chang H.Y. (2009). SIRT6 links histone H3 lysine 9 deacetylation to NF-kappaB-dependent gene expression and organismal life span. Cell.

[135] Kawahara T.L., Rapicavoli N.A., Wu A.R., Qu K., Quake S.R., Chang H.Y. (2011). Dynamic chromatin localization of Sirt6 shapes stress- and aging-related transcriptional networks. PLoS Genet..

[136] Schug T.T., Xu Q., Gao H., Peres-da-Silva A., Draper D.W., Fessler M.B., Purushotham A., Li X. (2010). Myeloid deletion of SIRT1 induces inflammatory signaling in response to environmental stress. Mol. Cell. Biol..

[137] Schneider K.S., Chan J.Y. (2013). Emerging role of Nrf2 in adipocytes and adipose biology. Adv. Nutr..

[138] Zhang Z., Zhou S., Jiang X., Wang Y.H., Li F., Wang Y.G., Zheng Y., Cai L. (2015). The role of the Nrf2/Keap1 pathway in obesity and metabolic syndrome. Rev. Endocr. Metab. Disord..

[139] Pi J., Leung L., Xue P., Wang W., Hou Y., Liu D., Yehuda-Shnaidman E., Lee C., Lau J., Kurtz T.W. (2010). Deficiency in the nuclear factor E2-related factor-2 transcription factor results in impaired adipogenesis and protects against diet-induced obesity. J. Biol. Chem..

[140] Vomhof-DeKrey E.E., Picklo M.J. (2012). NAD(P)H:quinone oxidoreductase 1 activity reduces hypertrophy in 3T3-L1 adipocytes. Free Radic. Biol. Med..

[141] Vanella L., Sanford C., Kim D.H., Abraham N.G., Ebraheim N. (2012). Oxidative stress and heme oxygenase-1 regulated human mesenchymal stem cells differentiation. Int. J. Hypertens..

[142] Kim B.R., Lee G.Y., Yu H., Maeng H.J., Oh T.J., Kim K.M., Moon J.H., Lim S., Jang H.C., Choi S.H. (2018). Suppression of Nrf2 attenuates adipogenesis and decreases FGF21 expression through PPAR gamma in 3T3-L1 cells. Biochem. Biophys. Res. Commun..

[143] Costanzo M., Boschi F., Carton F., Conti G., Covi V., Tabaracci G., Sbarbati A., Malatesta M. (2018). Low ozone concentrations promote adipogenesis in human adipose-derived adult stem cells. Eur. J. Histochem..

[144] Cisterna B., Costanzo M., Boschi F., Carton F., Covi V., Tabaracci G., Malatesta M. (2019). Exploring the potential of mild ozonisation in adipose tissue regeneration and differentiation. Eur. J. Histochem..

[145] Rojo de la Vega M., Chapman E., Zhang D.D. (2018). NRF2 and the Hallmarks of Cancer. Cancer Cell.

[146] Menegon S., Columbano A., Giordano S. (2016). The Dual Roles of NRF2 in Cancer. Trends Mol. Med..

[147] DeNicola G.M., Karreth F.A., Humpton T.J., Gopinathan A., Wei C., Frese K., Mangal D., Yu K.H., Yeo C.J., Calhoun E.S. (2011). Oncogene-induced Nrf2 transcription promotes ROS detoxification and tumorigenesis. Nature.

[148] Mitsuishi Y., Taguchi K., Kawatani Y., Shibata T., Nukiwa T., Aburatani H., Yamamoto M., Motohashi H. (2012). Nrf2 redirects glucose and glutamine into anabolic pathways in metabolic reprogramming. Cancer Cell.

[149] Chio I.I., Jafarnejad S.M., Ponz-Sarvise M., Park Y., Rivera K., Palm W., Wilson J., Sangar V., Hao Y., Öhlund D. (2016). NRF2 Promotes Tumor Maintenance by Modulating mRNA Translation in Pancreatic Cancer. Cell.

[150] Hojo T., Maishi N., Towfik A.M., Akiyama K., Ohga N., Shindoh M., Hida Y., Minowa K., Fujisawa T., Hida K. (2017). ROS enhance angiogenic properties via regulation of NRF2 in tumor endothelial cells. Oncotarget.

[151] Kang K.A., Hyun J.W. (2017). Oxidative Stress, Nrf2, and Epigenetic Modification Contribute to Anticancer Drug Resistance. Toxicol. Res..

[152] Petrucci M.T., Gallucci C., Agrillo A., Mustazza M.C., Foà R. (2007). Role of ozone therapy in the treatment of osteonecrosis of the jaws in multiple myeloma patients. Haematologica.

[153] Clavo B., Santana-Rodriguez N., Llontop P., Gutierrez D., Ceballos D., Méndez C., Rovira G., Suarez G., Rey-Baltar D., Garcia-Cabrera L. (2015). Ozone Therapy in the Management of Persistent Radiation-Induced Rectal Bleeding in Prostate Cancer Patients. Evid Based Complement. Alternat. Med..

[154] Motohashi H., Yamamoto M. (2004). Nrf2-Keap1 defines a physiologically important stress response mechanism. Trends Mol. Med..

[155] Cuadrado A., Rojo A.I., Wells G., Hayes J.D., Cousin S.P., Rumsey W.L., Attucks O.C., Franklin S., Levonen A.L., Kensler T.W. (2019). Therapeutic targeting of the NRF2 and KEAP1 partnership in chronic diseases. Nat. Rev. Drug Discov..

[156] Cuadrado A., Manda G., Hassan A., Alcaraz M.J., Barbas C., Daiber A., Ghezzi P., León R., López M.G., Oliva B. (2018). Transcription Factor NRF2 as a Therapeutic Target for Chronic Diseases: A Systems Medicine Approach. Pharm. Rev..

